# Association of *BRCA1/2*defects with genomic scores predictive of DNA damage repair deficiency among breast cancer subtypes

**DOI:** 10.1186/s13058-014-0475-x

**Published:** 2014-12-05

**Authors:** Kirsten M Timms, Victor Abkevich, Elisha Hughes, Chris Neff, Julia Reid, Brian Morris, Saritha Kalva, Jennifer Potter, Thanh V Tran, Jian Chen, Diana Iliev, Zaina Sangale, Eliso Tikishvili, Michael Perry, Andrey Zharkikh, Alexander Gutin, Jerry S Lanchbury

**Affiliations:** Myriad Genetics Inc., 320 Wakara Way, Salt Lake City, 84108 UT USA

## Abstract

**Introduction:**

Homologous recombination (HR) DNA repair is of clinical relevance in breast cancer. Three DNA-based homologous recombination deficiency (HRD) scores (HRD-loss of heterozygosity score (LOH), HRD-telomeric allelic imbalance score (TAI), and HRD-large-scale state transition score (LST)) have been developed that are highly correlated with defects in *BRCA1/2*, and are associated with response to platinum therapy in triple negative breast and ovarian cancer. This study examines the frequency of *BRCA1/2* defects among different breast cancer subtypes, and the ability of the HRD scores to identify breast tumors with defects in the homologous recombination DNA repair pathway.

**Methods:**

215 breast tumors representing all *ER/HER2* subtypes were obtained from commercial vendors. Next-generation sequencing based assays were used to generate genome wide SNP profiles, *BRCA1/2* mutation screening, and *BRCA1* promoter methylation data.

**Results:**

*BRCA1/2* deleterious mutations were observed in all breast cancer subtypes. *BRCA1* promoter methylation was observed almost exclusively in triple negative breast cancer. *BRCA1/2* deficient tumors were identified with *BRCA1*/2 mutations, or *BRCA1* promoter methylation, and loss of the second allele of the affected gene. All three HRD scores were highly associated with *BRCA1/2* deficiency (HRD-LOH: *P* = 1.3 × 10^-17^; HRD-TAI: *P* = 1.5 × 10^-19^; HRD-LST: *P* = 3.5 × 10^-18^). A combined score (HRD-mean) was calculated using the arithmetic mean of the three scores. In multivariable analyses the HRD-mean score captured significant *BRCA1/2* deficiency information not captured by the three individual scores, or by clinical variables (*P* values for HRD-Mean adjusted for HRD-LOH: *P* = 1.4 × 10^-8^; HRD-TAI: *P* = 2.9 × 10^-7^; HRD-LST: *P* = 2.8 × 10^-8^; clinical variables: *P* = 1.2 × 10^-16^).

**Conclusions:**

The HRD scores showed strong correlation with *BRCA1/2* deficiency regardless of breast cancer subtype. The frequency of elevated scores suggests that a significant proportion of all breast tumor subtypes may carry defects in the homologous recombination DNA repair pathway. The HRD scores can be combined to produce a more robust predictor of HRD. The combination of a robust score, and the FFPE compatible assay described in this study, may facilitate use of agents targeting homologous recombination DNA repair in the clinical setting.

**Electronic supplementary material:**

The online version of this article (doi:10.1186/s13058-014-0475-x) contains supplementary material, which is available to authorized users.

## Introduction

Defects in genes in the homologous recombination (HR) pathway are of potential therapeutic relevance in a variety of cancers. Clinical studies have demonstrated that *BRCA1/2*-deficient tumors are sensitive to both platinum salts and poly(ADP-ribose) polymerase inhibitors [1],[2]. *BRCA1/2* deficiency is known to be a result of deleterious germline or somatic mutations, or methylation of the *BRCA1* promoter. Numerous studies have investigated the rate of *BRCA1/2* mutations in triple-negative breast cancer (TNBC), with reported mutation rates ranging from 10 to 40% in this breast cancer subtype [3]-[8]. Many of these studies, however, focused on select patient populations known to be enriched for *BRCA1/2* mutations. Methylation of the *BRCA1* promoter and associated loss of expression of the gene have been reported in approximately 25% of breast cancers, with the frequency in TNBC reported to be as high as 31% [9]. These studies suggest that the frequency of *BRCA1/2* deficiency in TNBC is between 45 and 70%. In light of this, current clinical studies are focused on investigating TNBC for response to agents that are believed to exploit HR defects, including platinum agents and poly (ADP-ribose) polymerase inhibitors.

Genomic instability as a consequence of double-stranded DNA repair deficiency is a hallmark of TNBC [10]. Recently, three quantitative metrics accurately reflecting this genomic instability have been developed, namely whole genome tumor loss of heterozygosity profiles (homologous recombination deficiency–loss of heterozygosity (HRD-LOH) score) [11], telomeric allelic imbalance (homologous recombination deficiency–telomeric allelic imbalance (HRD-TAI) score) [12], and large-scale state transitions (homologous recombination deficiency– large-scale state transition (HRD-LST) score) [13] All three scores are highly correlated with defects in *BRCA1/2* and other HR pathway genes in breast cancer or ovarian cancer, and are associated with sensitivity to platinum agents [11]-[14].

While the role of *BRCA1/2* defects has been well studied in TNBC, significantly less information is available for other breast cancer subtypes. Even less is known about the proportion of non-TNBC tumors with elevated HRD-LOH, HRD-TAI, or HRD-LST scores reflecting loss of double-stranded DNA break repair capacity. This study examines the frequency of *BRCA1/2* defects and elevated scores across breast cancer subtypes, and examines the association of the HRD-LOH, HRD-TAI, and HRD-LST scores with *BRCA1/2* deficiency in breast tumors.

We also report the development of a next-generation sequencing-based assay that can be used to calculate all three scores, and is compatible with DNA extracted from formalin-fixed paraffin-embedded (FFPE)-treated tumor samples. Development of this assay should facilitate the use of these scores in the clinical setting.

## Materials and methods

### Breast tumor samples

Two hundred and fifteen breast tumor samples, and matched normal tissue blocks from the same patient, were obtained from four commercial vendors (Asterand, Detroit, MI, USA; ILSBio, Chestertown, MD, USA; ProteoGenex, Culver City, CA, USA; Indivumed, Hamburg, Germany). Samples were selected at random from the inventory list provided by each vendor, with the exception that attempts were made to balance the number of samples from each breast cancer subtype as defined by estrogen receptor (*ER*) and tyrosine kinase-type cell surface receptor *HER2* status (triple-negative, *n* = 63; *ER* ^+^/*HER2* ^–^, *n* = 51; *ER* ^–^/*HER2* ^+^, *n* = 38; *ER* ^+^/*HER2* ^+^, *n* = 63). The vendors provided the results of immunohistochemistry *ER* and *HER2* analysis. All samples were obtained under Institutional Review Board-approved protocols. Patient and tumor characteristics are shown in Tables S1 and S2 in Additional file 1.

### Extraction of DNA from frozen tumors

A 5 μm hematoxylin and eosin slide was created and reviewed by a pathologist to facilitate enrichment of tumor-derived DNA. Frozen 10 μm sections were cut and the regions of highest tumor cell density were scraped from the slide. The Promega Maxwell 16 LEV Blood DNA kit (AS1290; Promega, Madison, WI, USA) was used to extract DNA. Tissue samples were incubated overnight at 56°C with proteinase K and lysis buffer in a shaking heat block. After the overnight incubation, undigested material was spun out and the Maxwell cartridges were loaded. Genomic DNA was eluted in 60 μl water.

### Promoter methylation quantitative PCR assays

The Methyl-Profiler DNA Methylation PCR Assay System (SABiosciences, Valencia, CA, USA) was used to quantify methylation levels. The assay was performed as per the manufacturer’s recommended protocol. A description of the assay is provided in [11].

### Hybridization capture and sequencing

A custom enrichment panel was developed that targeted 54,091 single nucleotide polymorphisms (SNPs) distributed across the complete human genome (Agilent Technologies, Santa Clara, CA, USA). A detailed description of the panel design and development is provided in Materials and Methods in Additional file 1. The final panel also included an additional 685 probes targeting the complete coding region of *BRCA1* and *BRCA2*. A detailed description of the assay process is also provided in Materials and Methods in Additional file 1.

### *BRCA1* and *BRCA2*mutation screening

Variant and large rearrangement detection was performed on sequences from *BRCA1* and *BRCA2*. Complete descriptions of the sequence alignment and mutation detection methods are provided in Materials and Methods in Additional file 1.

Mutations identified were only included in the analysis if classified as deleterious or suspected deleterious based on previously described criteria [15].

### Calculation of HRD-LOH, HRD-TAI, and HRD-LST scores

The allele specific copy number at each SNP location was determined using an algorithm described in [11]. Descriptions of the homologous recombination deficiency (HRD) scores are provided in Materials and Methods in Additional file 1.

### Selection of samples for statistical analysis

Eighteen of 215 samples were excluded from the statistical analysis due to either high levels of contamination with noncancerous cells and/or low-quality SNP data (Materials and Methods in Additional file 1).

### Statistical analysis

Descriptions of the statistical analyses performed are provided in Materials and Methods in Additional file 1.

## Results

### Frequency of *BRCA1/2*defects

*BRCA1* and *BRCA2* sequence data were used to screen for both somatic and germline deleterious mutations. The analysis included detection of short variants and longer rearrangements spanning up to multiple exons in length. Variant analysis was successfully performed on 100% of samples, while large rearrangement analysis proved less robust, with 167/215 samples producing data that passed the quality control metrics for this assay. Deleterious mutations were observed in 25/215 individuals. Subsequently, DNA extracted from matching normal tissue samples from 24 out of the 25 individuals was used to determine whether the mutations found were germline or somatic. Four of the mutation carriers (16%) had multiple deleterious variants. Sequencing of matched normal DNA from these patients confirmed that each patient carried only one germline deleterious mutation and therefore the additional mutations in these individuals were somatic. In those individuals with a single deleterious mutation, loss of heterozygosity was observed at the affected gene in all but one individual. One individual was identified who carried a somatic *BRCA1* deleterious mutation (Y1522X) in one allele; this individual had a total of three copies of *BRCA1*, two of which appeared to be fully functional. Therefore, for the purposes of further analysis we classified this individual as *BRCA1/2* intact. Results of the mutation screening analysis are shown in Tables S3 and S4 in Additional file 1.

As expected, the highest rates of *BRCA1/2* mutations were observed in triple-negative tumors (15.9%). In one TNBC patient, the only *BRCA1* mutation was somatic; the other six individuals with a single deleterious mutation all carried germline mutations. Normal tissue DNA was not available for one TNBC patient carrying a deletion of one exon in *BRCA1* in their tumor sample. Consequently, it was not possible to determine whether this deleterious mutation was a germline mutation or a somatic mutation. Two additional TNBC tumors carried both germline and somatic mutations. *BRCA1/2* mutations were detected in all three of the other breast cancer subtypes with frequencies ranging from 7.8 to 11.1% (Table S3 in Additional file 1). The fraction of somatic mutations observed in these subtypes ranged from 0 to 50%; however, the total number of mutants was too low to obtain a reliable estimate of the frequency of germline versus somatic *BRCA1/2* mutations in the different subtypes.

Significant rates of *BRCA1* promoter methylation have been reported in triple-negative breast tumors. In this dataset, *BRCA1* promoter methylation was found almost exclusively in triple-negative tumors. Twenty-one percent of the triple-negative tumors showed significant levels of methylation of the *BRCA1* promoter, compared with <2% in each of the other breast cancer subtypes (Table S3 in Additional file 1). All tumors with *BRCA1* promoter methylation had confirmed loss of the second allele by loss of heterozygosity.

### HRD-LOH, HRD-TAI, and HRD-LST scores across breast cancer subtypes and association with *BRCA1/2*deficiency

A custom Agilent SureSelect hybridization enrichment assay was developed to enable the generation of high-quality copy number and allele dosage data from low-yield, low-quality FFPE-treated tumor samples. The development of this assay is described in detail in Materials and Methods in Additional file 1. Improved performance of the SNP sequencing assay on both frozen and FFPE tumor samples compared with SNP microarray data is shown in Additional file 2. SNP data were used to calculate HRD-LOH, HRD-TAI, and HRD-LST scores. One hundred and ninety-seven of 215 samples gave scores that passed the quality control criteria used. Thirty-eight of these samples were *BRCA1/2* deficient. In univariate logistic regression analysis, each of the HRD scores (HRD-LOH, HRD-TAI, and HRD-LST) was significantly associated with *BRCA1/2* deficiency (Table 1). Univariate results for age at diagnosis, breast cancer subtype, and tumor grade (both categorical and numeric) were also statistically significant (Table 1). Cancer stage was not associated with *BRCA1/2* status.

**Table 1:** **Results from univariate logistic regression with HRD scores or clinical variables as predictors of** ***BRCA1/2*** **deficiency**

	Number of patients	Number *BRCA1* / *2* deficient	*P* value	OR (95% CI)
HRD-LOH score	197	38	1.3 × 10^-17^	22 (8.4, 58)
HRD-TAI score	197	38	1.5 × 10^-19^	17 (7.2, 41)
HRD-LST score	197	38	3.5 × 10^-18^	19 (7.7, 46)
HRD-mean score	197	38	1.1 × 10^-24^	90 (22, 360)
Age at diagnosis	196	37	0.0071	0.96 (0.94, 0.99)
Stage			0.88	
I	13	3		1
II	121	23		0.78 (0.2, 3.1)
III	54	9		0.67 (0.15, 2.9)
IV	3	1		1.7 (0.11, 25)
Cancer subtype			1.2 × 10^-5^	
TNBC	52	23		8.5 (2.3, 31)
*ER* ^–^/*HER2* ^+^	35	3		1
*ER* ^+^/*HER2* ^–^	50	5		1.2 (0.26, 5.3)
*ER* ^+^/*HER2* ^+^	60	7		1.4 (0.34, 5.8)
Grade^a^ (categorical)			0.0011	NA
1	17	0		1
2	102	14		∞ (0, ∞)
3	71	21		∞ (0, ∞)
Grade (numerical)			0.00053	3.1 (1.6, 6.3)

Odds ratios for HRD scores are reported per interquartile range of the score. The odds ratio for age is reported per year. The odds ratio for grade (numerical) is per unit. CI, confidence interval; *ER*, estrogen receptor; *HER2*, tyrosine kinase-type cell surface receptor *HER2*; HRD, homologous recombination deficiency; HRD-LOH, homologous recombination deficiency-loss of heterozygosity; HRD-LST, homologous recombination deficiency-large-scale state transition; HRD-Mean, mean of the three HRD scores; HRD-TAI, homologous recombination deficiency-telomeric allelic imbalance; NA, not available; OR, odds ratio; TNBC, triple-negative breast cancer. ^a^The odds ratio for categorical grade is inestimable due to zero *BRCA1*/*2*-deficient grade 1 tumors.

**Figure 1:**
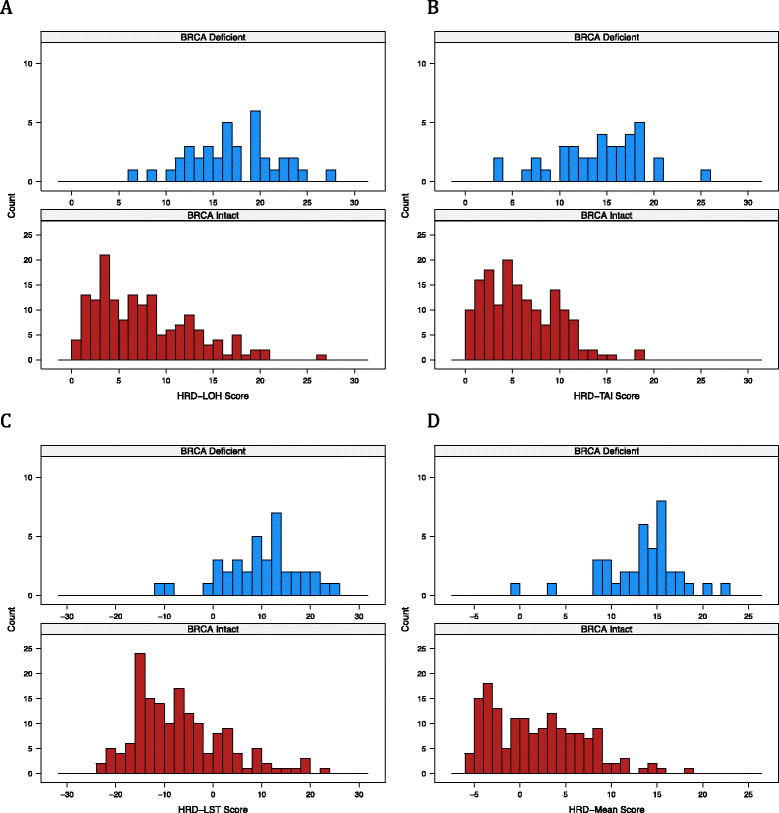
**Distribution of scores in** ***BRCA1/2*** -**intact and** ***BRCA1/2*** -**deficient samples. (A)** Homologous recombination deficiency-loss of heterozygosity (HRD-LOH) score. **(B)** Homologous recombination deficiency-telomeric allelic imbalance (HRD-TAI) score. **(C)** Homologous recombination deficiency-large-scale state transition (HRD-LST) score. **(D)** Mean of the homologous recombination deficiency (HRD-Mean) score. Red bars, *BRCA1/2*-intact samples; blue bars, *BRCA1/2*-deficient samples.

Fifty-two of 197 samples were from TNBC samples, including 23 that were *BRCA1/2* deficient. Each of the HRD scores was significantly associated with *BRCA1/2* deficiency in this breast cancer subtype (Table S5 in Additional file 1). When the same analysis was performed for each individual subtype, significant association is also seen for all scores except in the *ER* ^–^/*HER2* ^+^ subtype (Table S5 in Additional file 1). The distribution of scores is shown for *BRCA1/2*-deficient versus *BRCA1/2*-intact samples in Figure 1. High HRD scores were observed in *BRCA1/2*-intact tumors, in addition to *BRCA1/2*-deficient tumors, suggesting that a subset of these tumors have HR deficiency via some alternate mechanism.

**Figure 2:**
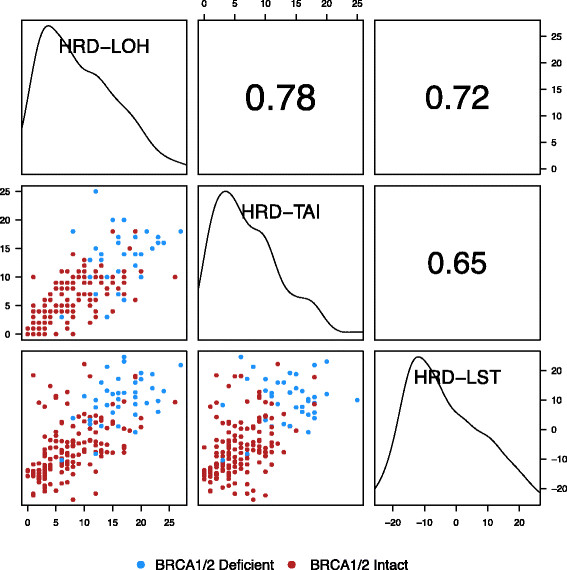
**Spearman correlation of three different homologous recombination deficiency scores.** Panels above the diagonal show correlation. Diagonal panels show density plots. HRD-LOH, homologous recombination deficiency-loss of heterozygosity; HRD-LST, homologous recombination deficiency-large-scale state transition; HRD-TAI, homologous recombination deficiency-telomeric allelic imbalance.

Pairwise correlations of the HRD-LOH, HRD-TAI, and HRD-LST scores were examined graphically, and quantified with Spearman rank-sum correlation. All pairwise correlations of scores showed positive correlation significantly different from zero (*P* < 10^-16^) (Figure 2).

Table 2 presents the results of a three-term multivariable logistic regression including HRD-LOH, HRD-TAI, and HRD-LST scores as predictors of *BRCA1/2* deficiency. In this analysis, both HRD-TAI (*P* = 0.00016) and HRD-LST (*P* = 0.00014) scores captured significant *BRCA1/2* deficiency information independent of the other scores. The HRD-LOH score did not reach statistical significance (*P* = 0.069).

**Table 2:** **Results from a three-term multivariable logistic regression model with HRD-LOH, HRD-TAI, and HRD-LST scores as predictors of** ***BRCA1/2*** **deficiency**

Score	*P* value	OR (95% CI)
HRD-LOH	0.069	3.0 (0.89, 9.8)
HRD-TAI	0.00016	5.8 (2.1, 16)
HRD-LST	0.00014	7.4 (2.4, 23)

Odds ratios are reported per interquartile range. This analysis included 197 patients, 38 of whom were *BRCA1* or *BRCA2* deficient. CI, confidence interval; HRD-LOH, homologous recombination deficiency-loss of heterozygosity; HRD-LST, homologous recombination deficiency-large-scale state transition; HRD-TAI, homologous recombination deficiency-telomeric allelic imbalance; OR, odds ratio.

Since each of the HRD measures appears to offer additional information that could be potentially clinically useful, we explored the utility of combining the three scores. Our first approach was to combine the HRD scores according to the best linear combination from a logistic regression model to predict *BRCA1/2* deficiency in this dataset. The resulting model-based score was:

HRD‐model=0.11xHRD‐LOH+0.25xHRD‐TAI+0.12xHRD‐LST

A disadvantage of this approach is that the optimal weights for individual HRD scores are overfitted because of the relatively small size of the dataset and moderate correlations between the scores.

An alternate approach is to consider a simple arithmetic mean of the three scores (HRD-Mean score). A rationale for averaging the scores is that all three scores essentially count events, breakpoints, or regions between breakpoints associated with HR deficiency. Moreover, according to univariate association of the scores with *BRCA1/2* deficiency (Table 1), they seem to capture relevant events equally well.

To assess whether the HRD-Mean score adequately captured the *BRCA1/2* deficiency information of its three components, we tested three bivariate logistic regression models. Each included the HRD-Mean score and one of the three individual scores. None of the component scores added significantly to the HRD-Mean score at the 5% significance level (HRD-LOH, *P* = 0.89; HRD-TAI, *P* = 0.09; HRD-LST, *P* = 0.28). In contrast, in each of the bivariate models’ HRD-Mean score added significant information to the individual scores (*P* = 1.4 × 10^-8^ for HRD-LOH; *P* = 2.9 × 10^-7^ for HRD-TAI; *P* = 2.8 × 10^-8^ for HRD-LST).

The HRD-Mean score was also compared with the HRD model score. In a bivariate logistic regression model, the HRD model score did not add significant independent *BRCA1/2* deficiency information to the HRD-Mean score (*P* = 0.089).

**Figure 3:**
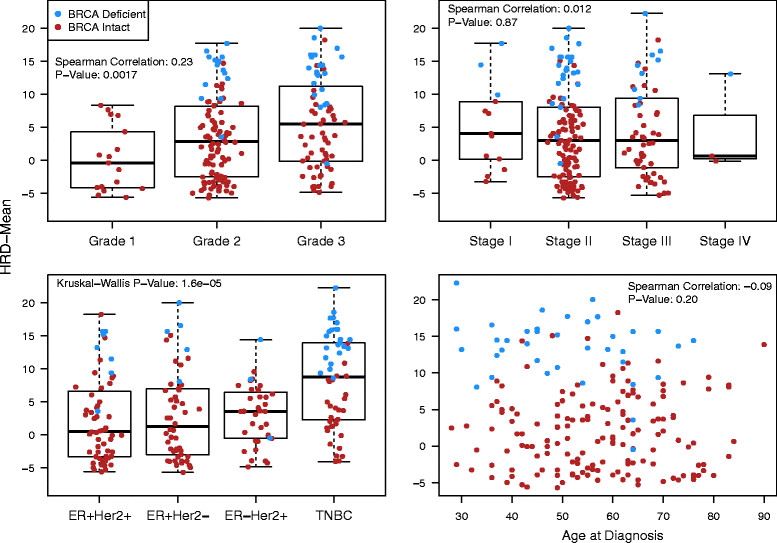
**Association of clinical variables with the HRD-Mean score.** Upper left panel: tumor grade; upper right panel: tumor stage; lower left panel: tumor IHC subtype; lower right panel: age at diagnosis. Mean of the homologous recombination deficiency-loss of heterozygosity, homologous recombination deficiency-large-scale state transition, and homologous recombination deficiency-telomeric allelic imbalance scores (HRD-Mean score). *ER*, estrogen receptor; *HER2*, tyrosine kinase-type cell surface receptor *HER2*; HRD, homologous recombination deficiency; TNBC, triple-negative breast cancer.

Data for tumor stage and grade and for age at diagnosis were available for the majority of samples (Table S1 in Additional file 1). Associations of these clinical variables with the HRD-Mean score are shown in Figure 3. The HRD-Mean score was not significantly correlated with tumor stage or age at diagnosis at the 5% confidence level, but there was significant correlation with tumor grade (Spearman correlation 0.23, *P* = 0.0017). The HRD-Mean score also differed significantly among breast cancer subtypes (*P* = 1.6 × 10^-5^) according to a Kruskal–Wallis one-way analysis of variance test.

Heterogeneity of the HRD-Mean score among clinical breast cancer subpopulations was examined in multivariable logistic regression models. For each clinical variable we added a term for the interaction with the HRD-Mean score to a model including all clinical variables and the HRD-Mean score. None of the interaction terms reached significance at the 5% significance level. We therefore found no evidence to suggest that the probability of *BRCA1/2* deficiency conferred by the HRD-Mean score varies among clinical subpopulations.

Multivariable analysis was used to examine a model based on the HRD-Mean score and all available clinical variables. The HRD-Mean score captured significant *BRCA1/2* deficiency information that was not captured by clinical variables (*P* = 1.2 × 10^-16^). Of the available clinical variables, only age at diagnosis maintained significance in the multivariable setting (*P* = 0.027) (Table 3).

**Table 3:** **Results from multivariable logistic regression of** ***BRCA1/2*** **deficiency**

	Number of patients	Number *BRCA1* / *2* deficient	*P* value	OR (95% CI)
HRD-Mean score	186	34	1.2 × 10^-16^	87 (17, 450)
Age at diagnosis	186	34	0.027	0.95 (0.91, 1.0)
Stage			0.63	
I	12	3		1
II	119	22		2.4 (0.22, 27)
III	52	8		0.99 (0.073, 13)
IV	3	1		3.1 (0.0011, 9100)
Grade^a^			0.40	
1	17	0		1
2	99	14		∞ (0, ∞)
3	70	20		∞ (0, ∞)
Subtype			0.087	
TNBC	44	19		3.9 (0.62, 24)
*ER* ^–^/*HER2* ^+^	34	3		1
*ER* ^+^/*HER2* ^–^	48	5		0.39 (0.039, 3.8)
*ER* ^+^/*HER2* ^+^	60	7		1.3 (0.16, 10)

Odds ratio for the HRD-Mean score is reported per interquartile range of the score. The odds ratio for age at diagnosis is reported per year. This analysis includes 186 patients with complete clinical records. Thirty-four patients were *BRCA1* or *BRCA2* deficient. CI, confidence interval; *ER*, estrogen receptor; *HER2*, tyrosine kinase-type cell surface receptor *HER2*; HRD-Mean, mean of the homologous recombination deficiency-loss of heterozygosity, homologous recombination deficiency-large-scale state transition, and homologous recombination deficiency-telomeric allelic imbalance scores; OR, odds ratio; TNBC, triple-negative breast cancer. ^a^The odds ratio for grade is inestimable due to zero *BRCA1/2*-deficient grade 1 tumors.

To assess the robustness of the hybridization enrichment assay, 42 samples were run in duplicate. Both sets of data were analyzed separately in a blinded analysis. The Pearson correlation coefficient was 0.89 for the HRD-LOH score, 0.92 for the HRD-TAI score, 0.84 for the HRD-LST score, and 0.93 for the HRD-Mean score (Additional file 3).

## Discussion

Defects in homologous recombination, including *BRCA1/2* defects, are a relatively frequent event in TNBC, and the exploitation of these defects in the clinical setting is under intensive study. Less is known about the frequency of homologous recombination defects in other subtypes of breast cancer. In this study, the frequency of *BRCA1/2* mutations ranged from ~8 to ~16% across four subtypes of breast cancer as defined by immunohistochemistry subtyping. Sequencing of matched tumor and normal DNA samples determined that 75% of *BRCA1/2* mutant tumors carried a germline *BRCA1/2* mutation. The most common method for loss of the second allele was via loss of heterozygosity; however, 17% of the tumors did not have loss of heterozygosity but carried somatic deleterious mutations in addition to their germline mutation. In addition, an apparently sporadic breast tumor was seen in one individual carrying a *BRCA2* somatic deleterious mutation. *BRCA1/2* deficiency, including deleterious mutations and *BRCA1* promoter methylation, ranged from ~10 to ~37% across the four subtypes.

Three independent studies recently reported the development of DNA-based genomic scores that were associated with defects in *BRCA1/2* [11]-[13]. In addition, all three scores have been shown to either predict sensitivity to platinum drugs *in vitro*, and/or pathologic response to neoadjuvant platinum treatment [12]-[14].

All three scores showed strong correlation with *BRCA1/2* deficiency regardless of subtype, and the frequency of elevated scores suggests that a significant proportion of all breast tumor subtypes carry defects in the homologous recombination DNA repair pathway. These findings suggest that agents which target or exploit DNA damage repair may prove effective across a subset of tumors from all subtypes of breast cancer.

All three scores were significantly correlated with one another, suggesting that they all measure the same core genomic phenomenon. However, logistic regression analysis indicates that the scores could be combined resulting in stronger association with *BRCA1/2* deficiency in this dataset. Further study is required to define a robust optimal model for response to clinical agents in an adequately powered dataset. In this study, an optimized model (HRD model) did not capture additional *BRCA1/2* deficiency information when compared with the arithmetic mean (HRD-Mean score) of the three scores.

Implementation of these scores, either singly or in combination, in the clinical setting requires an assay that is compatible with core needle biopsies that have been formalin fixed and paraffin embedded. Samples of this type yield very low-quantity and low-quality DNA. DNA extracted from these FFPE-treated samples does not perform well in SNP microarray analysis.

Liquid hybridization-based target enrichment technologies have been developed for production of libraries for next-generation sequencing. These methodologies enable targeted sequencing of regions of interest after reduction in genomic complexity, resulting in decreased sequencing costs. Preliminary tests indicated that the available assays should be compatible with DNA derived from FFPE DNA. In this study we report the development of a capture panel that targets ~54,000 SNPs distributed across the genome. Allele counts from the sequencing information this panel provides can be used for copy number and loss of heterozygosity reconstruction, and the calculation of all three of the HRD scores. In addition, *BRCA1* and *BRCA2* capture probes are included on the panel, which enable high-quality mutation screening for deleterious variants in these genes in the same assay.

## Conclusions

*BRCA1/2* mutations are most common in TNBC; however, both germline and somatic mutations were observed in all subtypes of breast cancer. In contrast, *BRCA1* promoter methylation was confined almost exclusively to TNBC. Elevated HRD scores were highly associated with *BRCA1/2* deficiency regardless of breast cancer subtype, and the most robust predictor of HRD deficiency is likely to be a combination of all three individual HRD scores. The combination of a robust score capable of identifying tumors with defects in homologous recombination DNA repair, and an assay compatible with FFPE clinical pathological specimens should facilitate the development and use of agents targeting double-stranded DNA damage repair in the clinical setting. In addition, these data suggest that such agents may have utility across all subtypes of breast cancer when combined with an appropriate biomarker.

## Additional files

## Electronic supplementary material

Additional file 1: **Detailed description of the** **Materials** **and** **Methods** **described in the manuscript, and Tables S1 to S5 showing data analysis not included in the main text.** (DOCX 30 KB)

Additional file 2: Figure S1.: Showing allele dosage data generated by SNP microarrays (upper panels) or custom hybridization enrichment followed by next-generation sequencing (lower panels). (A) Frozen samples; (B) FFPE samples. (PDF 2 MB)

Additional file 3: Figure S2.: Showing correlation between duplicate samples run on the Agilent SureSelect hybridization enrichment assay. Pearson correlation coefficient = 0.93 for HRD-Mean. (PDF 30 KB)

Below are the links to the authors’ original submitted files for images.

Authors’ original file for figure 1

Authors’ original file for figure 2

Authors’ original file for figure 3

## Abbreviations

*BRCA*: breast cancer gene
*ER*: estrogen receptor
FFPE: formalin-fixed paraffin-embedded
*HER2*: tyrosine kinase-type cell surface receptor *HER2*
HR: homologous recombination
HRD: homologous recombination deficiency
HRD-LOH: homologous recombination deficiency–loss of heterozygosity
HRD-LST: homologous recombination deficiency–large-scale state transition
HRD-Mean: mean of HRD-LOH, HRD-LST, and HRD-TAI scores
HRD-TAI: homologous recombination deficiency–telomeric allelic imbalance
SNP: single nucleotide polymorphism
TNBC: triple-negative breast cancer
