# Supplementary material for: Association of BRCA1/2defects with genomic scores predictive of DNA damage repair deficiency among breast cancer subtypes
Source: Breast Cancer Res. 2014 Dec 5;16:475. doi: 10.1186/s13058-014-0475-x (PMC4308910; doi:10.1186/s13058-014-0475-x)
Supplement: Supplementary file 1 — Additional file 1: Detailed description of the Materials and Methods described in the manuscript, and Tables S1 to S5 showing data analysis not included in the main text. (DOCX 30 KB) [file 13058_2014_475_MOESM1_ESM.docx]

Association of *BRCA1/2* Defects with Genomic Scores Predictive of DNA Damage Repair Deficiency Among Breast Cancer Subtypes

Additional Material

Kirsten M Timms*, Victor Abkevich (victor@myriad.com), Elisha Hughes (ehughes@myriad.com), Chris Neff (cneff@myriad.com), Julia Reid (jreid@myriad.com), Brian Morris (bmorris@myriad.com), Saritha Kalva (skalva@myriad.com), Jennifer Potter (jpotter@myriad.com), Thanh V Tran (thanh@myriad.com), Jian Chen (jchen@myriad.com), Diana Iliev (diana@myriad.com), Zaina Sangale (zsangale@myriad.com), Eliso Tikishvili (etikishv@myriad.com), Michael Perry (mperry@myriad.com), Andrey Zharkikh (zharkikh@myriad.com), Alexander Gutin (agutin@myriad.com), and Jerry S Lanchbury (jlanchbu@myriad.com).

Myriad Genetics Inc., Salt Lake City, UT, USA

- Corresponding Author: Myriad Genetics Inc., 320 Wakara Way, Salt Lake City, UT 84108.

**Additional Materials and Methods**

**Hybridization capture and sequencing**

The SNPs targeted by this panel were selected from a starting set of 2.5 million. All 2.5 million SNPs were submitted for custom probe design, with 1.4 million passing the probe design process. From this set of 1.4 million 110,000 were selected which passed the following selection criteria: 1. Y chromosome and mitochondrial SNPs were removed; 2. SNPs were removed which had minor allele frequencies <5% in Caucasians or <1% in 3 other races; 3. SNPs with significant deviation from Hardy-Weinberg equilibrium in any of 4 different races were removed; 4. 110,000 SNPs were selected from the remainder that had the highest allele frequency in Caucasians, covered the genome evenly, and were not in linkage disequilibrium in any of the four races where data was available. Two custom designed capture panels were then created containing probes targeting ~55,000 SNPs each. Each panel was then used for target enrichment of samples from 5 FFPE tumors and the resulting libraries were sequenced. The 54.091 SNPs with the most robust performance were then selected for inclusion in the final panel. Probes targeting *BRCA1* and *BRCA2* were densely tiled at 25 bp intervals to ensure complete sequence coverage of both genes. Each probe was replicated 5x on the panel, with the exception of probes covering small regions or areas with low capture efficiency where the replication level was increased to 11X.

The resulting custom panel was tested by running a selection of cell line, frozen tumor, and FFPE tumor DNAs that had previously been run on SNP microarrays. The resulting data was compared to look for concordance between the different methodologies, and also to assess performance of the sequencing based method. The sequence based assay proved to be superior to microarrays regardless of sample type, with much lower noise overall (additional figure 1).

500ng – 1000ng of genomic DNA (gDNA) was used for the SureSelect XT capture method.  Briefly gDNA was sheared on a Covaris E220 so that the peak size was between 150 and 200 nucleotides.  Amplification of adapter-ligated library preceded an overnight hybridization at 65 degrees Celsius with the SureSelect biotinylated RNA library baits.  Following hybridization between individual adapter-ligated libraries and the RNA library baits, index tags were added by amplification so that pooled barcoded samples could be run on the Illumina HiSeq2500 sequencer (Illumina, San Diego, CA).

Individual libraries were pooled depending on the desired sequencing coverage and type of sequencing run, e.g. Rapid Run mode and High Output mode. Generally 6 individual samples were pooled together for sequencing runs that underwent Rapid Run mode and 12 samples were pooled together for sequencing runs that underwent High Output mode.  Individual sample libraries were combined such that each index-tagged sample would be present in equimolar amounts in the pool.  For most purposes pools were made so that each library was at a final concentration of 10nM.  From here the standard Illumina Sequencing protocol was followed to denature and dilute the pooled libraries to 7pM for loading on Rapid and High Output flow cells.

***BRCA1* and *BRCA2* mutation screening**

Sequence reads generated on the HiSeq2500 are trimmed at both the start and end to remove low quality bases that could generate spurious variant calls. Sequence trimming was largely performed according to the BWA program’s trimming algorithm (Burrows and Wheeler, 1994; Li and Durbin, 2009). For more detail see <http://solexaqa.sourceforge.net/>. Phred value 20 was used as a threshold for trimming at the start of sequences and 30 for trimming at the end. These thresholds were derived empirically. It is expected that the sequence quality will deteriorate towards the end of a read, so we use a higher threshold at the end of sequences.

For each read an in-house implementation of the Burrow Wheeler Transform algorithm (Burrows and Wheeler, 1994) was executed which performs a search of all exons in our database to determine the matching exon for each read.

To call variants each read was aligned with the expected wildtype sequence of the exon. This alignment was a pairwise alignment performed by JAligner (http://jaligner.sourceforge.net/). Any differences represent variants. Variant calls from all reads for a sample were compiled in order to calculate the frequencies of all identified variants.

**Large Rearrangement Detection**

For large rearrangement detection the number of reads *N* that mapped back to each base was normalized (*N_norm_*) using the total number of mapped back reads across all genes and SNP locations. A median normalized read count value *N_med_* in a large set of samples was determined for each base. Centered normalized read counts, defined as *N_cent_=N_norm_/N_med_*, were reviewed to detect large rearrangements encompassing one or more exons. The CV of centered normalized read counts for the exon 11 (largest exon) of both *BRCA1* and *BRCA2* was determined. If CV was below 0.09, all detected rearrangements were called. If the CV was between 0.9-0.12,only rearrangement encompassing two or more exons were called. If the value exceeded 0.12 the sample was rejected as not being able to call.

**SNP Analysis**

SNP sequence database for mapping sequence reads was created by cutting from the whole genome (version 19) sequences of the SNPs with 400 bp flanks around the SNP positions. The combined sequence was indexed for the BWT search and checked for the repetitiveness by counting the number of copies with three or less mismatches for each 100-base segment of the sequence. The SNP probes with multiple occurrences in the genome were excluded from the analysis.

The mapping of the sequence reads to the SNP sequence database was performed by a proprietary program that implements the BWT algorithm. Each sequence read was considered mapped if it matched to the database sequence with 7 or less mismatches.

Sequences reads overlapping a SNP position were used to count the SNP alleles.

If both forward and reverse reads of the same clone overlap the SNP position and produce the same allele, only one count was applied for this clone. Clones where the forward and reverse reads produced different alleles were considered a sequencing error and were not counted. Clones with both forward and reverse reads not overlapping the SNP position were counted separately from clones with reads overlapping the SNP position.

The resulting read counts were used to reconstruct allele specific copy number (ASCN) at each SNP location using an algorithm described in Abkevich et al, 2012.

**Quality of ASCN reconstruction**

To evaluate the quality of ASCN reconstruction, a quality metric, KS quality, was introduced. Specifically, for each sample, all SNPs were separated in two two groups, first group containing all SNPs with allelic imbalance and second group containing all SNPs with equal numbers of copies of the two parental alleles. Allele dosage *d* at each SNP was transformed as follows: *d_tr_ = d* if *d<0.5* and *d_tr_=1-d* otherwise. KS quality was defined as

*KS quality = sqrt(N_1_N_2_/(N_1_+N_2_))max|F_1_(d_tr_)-F_2_(d_tr_)|*

where *N_1_* and *N_2_* are the numbers of SNPs in the two groups, *F_1_(d_tr_)* and *F_2_(d_tr_)* are empirical distributions of the transformed allele dosage in the two groups, and maximum is taken over transformed dosage values between 0 and 0.5. In essence, KS quality is measuring how different distributions of transformed dosages between SNPs with balanced and imbalanced alleles. The specific definition of KS quality is based on Kolmogorov-Smirnov statistic. High quality ASCN reconstruction is expected to produce high KS quality. Through visual inspection of about hundred samples, a cutoff value 12.7 for KS quality has been established. ASCN reconstrauctions with KS quality below this cutoff are considered as failed. There are two major reasons for failures: (1) high noise level in the sequence data and (2) low tumor content in a sample.

**Calculation of HRD-LOH, HRD-TAI, and HRD-LST scores**

HRD-LOH score was defined as the number of LOH regions longer than 15 Mb but shorter than the whole chromosome (Abkevich et al, 2012). HRD-LOH score has been shown to be associated with *BRCA1*, *BRCA2*, and *RAD51C* deficiency in 609 ovarian tumors (Abkevich et al, 2012).

HRD-TAI score was defined as the number of regions with allelic imbalance that extend to one of the subtelomeres but do not cross the centromere (Birkbak et al, 2012). A region was counted only if it encompassed a certain minimum number of SNPs (on average approximately 1.8 Mb). We tested for association of HRD-TAI score with *BRCA1*, *BRCA2*, and *RAD51C* deficiency in three datasets of 609 ovarian tumors (data not shown) and found the association to be more significant if the cutoff for the size of HRD-TAI regions was increased to 11 Mb. Therefore, a modified HRD-TAIm score was defined as the number of regions with allelic imbalance that (a) extend to one of the subtelomeres, (b) do not cross the centromere and (c) are longer than 11 Mb.

HRD-LST score is the number of break points between regions longer than 10 Mb after filtering out regions shorter than 3 Mb (Popova et al., 2012). Different cutoffs for HRD-LST score were introduced for “near-diploid” and “near-tetraploid” tumors to separate *BRCA1/2* intact and deficient samples. We tested for association of HRD-LST score with *BRCA1*, *BRCA2*, and *RAD51C* deficiency in three datasets of 609 ovarian tumors (data not shown). We also observed that HRD-LST score increases with ploidy both within intact and deficient samples. Instead of using ploidy-specific cutoffs, the HRD-LST score was modified by adjusting it by ploidy:

LSTm = LST – kP

where P is ploidy and k is a constant. Based on multivariate logistic regression analysis with deficiency as an outcome and HRD-LST and P as predictors, k = 15.5 provided the best separation between intact and deficient samples.

**Statistical analysis**

All analyses were conducted using R version 3.0.2 (R Core Team, 2013). All reported p values were two-sided. The statistical tools employed in this study include Spearman rank-sum correlation, Kruskal-Wallis one-way analysis of variance, and logistic regression.

For logistic regression modeling, HRD scores and age at diagnosis were coded as numeric variables. Breast cancer stage and subtype were coded as categorical variables. Grade was analyzed as both a numeric and categorical variable, but was categorical unless otherwise noted.

The p values reported for unvariate logistic regression models are based on the partial likelihood ratio. Multivariate p values are based on the partial likelihood ratio for change in deviance from a full model (which includes all relevant predictors) versus a reduced model (which includes all predictors except for the predictor being evaluated, and any interaction terms involving the predictor being evaluated). Odds ratios for RD scores are reported per interquartile range.

**References**

Burrows M, Wheeler D: **A block-sorting lossless data compression algorithm.** Technical report, Digital Equipment Corporation, Palo Alto, California; 1994.

Li H, Durbin R: **Fast and accurate short read alignment with Burrows-Wheeler Transform.** Bioinformatics, 2009, **25**:1754-60.

R Core Team: **R: A language and environment for statistical computing.** R Foundation for Statistical Computing, Vienna, Austria. URL: <http://www.R-project.org/>; 2013.

**Additional Tables**

Table S1: Patient and cancer characteristics for all tumors.

|  | **All Patients (%)** | **Triple Negative (%)** | **ER+/HER2- (%)** | **ER-/HER2+ (%)** | **ER+/HER2+ (%)** | ***BRCA1/2* Mutant (%)** | ***BRCA1/2* Deficient (%)** |
| --- | --- | --- | --- | --- | --- | --- | --- |
| **Total Patients** | 215 (100) | 63 (29) | 51 (24) | 38 (18) | 63 (29) | 25 (12) | 39 (18) |
| **Age at Diagnosis** |  |  |  |  |  |  |  |
| **Range** | 28-90 | 29-90 | 33-80 | 29-76 | 28-79 | 33-79 | 29-76 |
| **Median** | 55 | 55 | 62 | 54 | 53 | 55 | 49 |
| **%<60** | 59 | 61 | 47 | 63 | 63 | 64 | 70 |
| **Stage** |  |  |  |  |  |  |  |
| **I** | 15 (7) | 9 (14) | 2(4) | 1 (3) | 3 (5) | 2 (8) | 3 (8) |
| **II** | 131 (61 | 34 (54) | 31 (61) | 27 (71) | 39 (62) | 18 (72) | 23 (61) |
| **III** | 59 (27) | 11 (17) | 18 (35) | 9 (24) | 21 (33) | 5 (20) | 9 (24) |
| **IV** | 3 (1) | 3 (5) | 0 (0) | 0 (0) | 0 (0) | 0 (0) | 1 (3) |
| **Unknown** | 7 (3) | 6 (10) | 0 (0) | 1 (3) | 0 (0) | 0 (0) | 2 (5) |
| **Grade** |  |  |  |  |  |  |  |
| **1** | 17 (8) | 4 (6) | 8 (16) | 0 (0) | 5 (8) | 0 (0) | 0 (0) |
| **2** | 112 (52) | 21 (33) | 31 (61) | 16 (42) | 44 (70) | 11 (44) | 14 (37) |
| **3** | 76 (35) | 30 (48) | 10 (20) | 22 (58) | 14 (22) | 13 (52) | 21 (55) |
| **Unknown** | 10 (5) | 8 (13) | 2 (4) | 0 (0) | 0 (0) | 1 (4) | 3 (8) |

Table S2: Patient and cancer characteristics for tumors with passing HRD scores.

|  | **All Patients (%)** | **Triple Negative (%)** | **ER+/HER2- (%)** | **ER-/HER2+ (%)** | **ER+/HER2+ (%)** | ***BRCA1/2* Mutant* (%)** | ***BRCA1/2* Deficient** (%)** |
| --- | --- | --- | --- | --- | --- | --- | --- |
| **Total Patients** | 197 (100) | 52 (26) | 50 (25) | 35 (18) | 60 (30) | 24 (12) | 38 (19) |
| **Age at Diagnosis** |  |  |  |  |  |  |  |
| **Range** | 28-90 | 29-90 | 33-80 | 29-76 | 28-79 | 33-79 | 29-76 |
| **Median** | 56 | 54 | 62 | 55 | 54.5 | 55.5 | 49 |
| **% <60** | 57 | 61 | 46 | 60 | 62 | 62.5 | 70 |
| **Stage** |  |  |  |  |  |  |  |
| **I** | 13 (7) | 7 (13) | 2 (4) | 1 (3) | 3 (5) | 2 (8) | 3 (8) |
| **II** | 121 (61) | 28 (54) | 31 (62) | 25 (71) | 37 (62) | 17 (71) | 23 (61) |
| **III** | 54 (27) | 9 (17) | 17 (34) | 8 (23) | 20 (33) | 5 (21) | 9 (24) |
| **IV** | 3 (2) | 3 (6) | 0 (0) | 0 (0) | 0 (0) | 0 (0) | 1 (3) |
| **Unknown** | 6 (3) | 5 (10) | 0 (0) | 1 (3) | 0 (0) | 0 (0) | 2 (5) |
| **Grade** |  |  |  |  |  |  |  |
| **1** | 17 (9) | 4 (8) | 8 (16) | 0 (0) | 5 (8) | 0 (0) | 0 (0) |
| **2** | 102 (52) | 17 (33) | 30 (60) | 13 (37) | 42 (70) | 10 (42) | 14 (37) |
| **3** | 71 (36) | 26 (50) | 10 (20) | 22 (63) | 13 (22) | 13 (54) | 21 (55) |
| **Unknown** | 7 (4) | 5 (10) | 2 (4) | 0 (0) | 0 (0) | 1 (4) | 3 (8) |

* Carriers of germline or somatic deleterious mutations in *BRCA1/2*, and with confirmed loss of the second allele of the affected gene.

** Carriers of germline or somatic deleterious mutations in *BRCA1/2*, or *BRCA1* promoter methylation, and with confirmed loss of the second allele of the affected gene.Table S3: *BRCA1/2* mutations and *BRCA1* promoter methylation among breast cancer subtypes.

| **Subtype** | **n** | ***BRCA1* Mutations** | ***BRCA2* Mutations** | **Total Mutants (%)** | ***BRCA1* Promoter Methylation (%)** |
| --- | --- | --- | --- | --- | --- |
| **Triple Negative** | 63 | 10 | 3 | 10 (16) | 13 (21) |
| **ER+/HER2-** | 51 | 2 | 2 | 4 (8) | 1 (2) |
| **ER-/HER2+** | 38 | 3 | 1 | 4 (11) | 0 |
| **ER+/HER2+** | 63 | 8* | 1 | 7* (11) | 1 (2) |

* Includes one individual who still retains intact functional copies of *BRCA1*Table S4: Frequency of *BRCA1* vs. *BRCA2* and germline vs. somatic mutations by subtype. Data is available for 24 individuals of the 25 individuals identified with deleterious mutations.

| **Subtype** | **Tumor Mutation Profile** | **n** | **Germline n** | **Somatic n** |
| --- | --- | --- | --- | --- |
| **Triple Negative** | 1 *BRCA1* mutation | 6 | 5 | 1 |
|  | 1 *BRCA2* mutation | 1 | 1 | 0 |
|  | 2 *BRCA1* mutations | 1* | 1 | 1 |
|  | 1 *BRCA1* mutation and 2 *BRCA2* mutations | 1 | 1 (*BRCA2*) | 2 |
| **ER+/HER2-** | 1 *BRCA1* mutation | 2 | 2 | 0 |
|  | 1 *BRCA2* mutation | 2 | 2 | 0 |
| **ER-/HER2+** | 1 *BRCA1* mutation | 3 | 2 | 1 |
|  | 1 *BRCA2* mutation | 1 | 0 | 1 |
| **ER+/HER2+** | 1 *BRCA1* mutation | 4** | 1 | 3** |
|  | 2 *BRCA1* mutations | 2* | 2 | 2 |
|  | 1 *BRCA2* mutation | 1 | 1 | 0 |

* Each individual had 1 germline and 1 somatic mutation in *BRCA1*

** Includes one individual who still retains intact functional copies of *BRCA1*

Table S5: Mean HRD-LOH, HRD-TAI, or HRD-LST score in *BRCA1/2* deficient and *BRCA1/2* intact tumors from each of 4 breast cancer subtypes.

|  | **Subtype** | **All** | **TNBC** | **ER+/HER2-** | **ER-/HER2+** | **ER+/HER2+** |
| --- | --- | --- | --- | --- | --- | --- |
|  | **Number of individuals** | 197 | 52 | 50 | 35 | 60 |
|  | **Number *BRCA1/2* deficient (%)** | 38 (100) | 23 (61) | 5 (13) | 3 (8) | 7 (18) |
| **HRD-LOH** | ***BRCA1/2* intact mean** | 7.2 | 8.2 | 7.1 | 8.3 | 6.0 |
|  | ***BRCA1/2* deficient mean** | 16.5 | 17.7 | 17.2 | 12.0 | 14.1 |
|  | **p value** | 1.3 x 10-17 | 1.5 x 10-8 | 0.0025 | 0.18 | 2.1 x 10-5 |
| **HRD-TAI** | ***BRCA1/2* intact mean** | 5.5 | 6.8 | 4.3 | 6.4 | 5.1 |
|  | ***BRCA1/2* deficient mean** | 13.7 | 13.5 | 15.0 | 7.7 | 15.9 |
|  | **p value** | 1.5 x 10-19 | 2.2 x 10-7 | 1.3 x 10-5 | 0.58 | 14 x 10-6 |
| **HRD-LST** | ***BRCA1/2* intact mean** | -7.0 | -5.1 | -6.7 | -6.7 | -8.3 |
|  | ***BRCA1/2* deficient mean** | 10.2 | 12.0 | 11.7 | 2.7 | 6.1 |
|  | **p value** | 3.5 x 10-18 | 8.0 x 10-11 | 3.2 x 10-4 | 0.082 | 0.0024 |
| **HRD-Mean** | ***BRCA1/2* intact mean** | 1.9 | 3.3 | 1.6 | 2.7 | 0.9 |
|  | ***BRCA1/2* deficient mean** | 13.4 | 14.4 | 14.6 | 7.5 | 12.0 |
|  | **p value** | 1.1 x 10-24 | 7.8 x 10-13 | 2.3 x 10-5 | 0.072 | 2.1 x 10-5 |
