# Supplementary figures and images for: Association of BRCA1/2defects with genomic scores predictive of DNA damage repair deficiency among breast cancer subtypes
Source: Breast Cancer Res. 2014 Dec 5;16:475. doi: 10.1186/s13058-014-0475-x (PMC4308910; doi:10.1186/s13058-014-0475-x)

A:

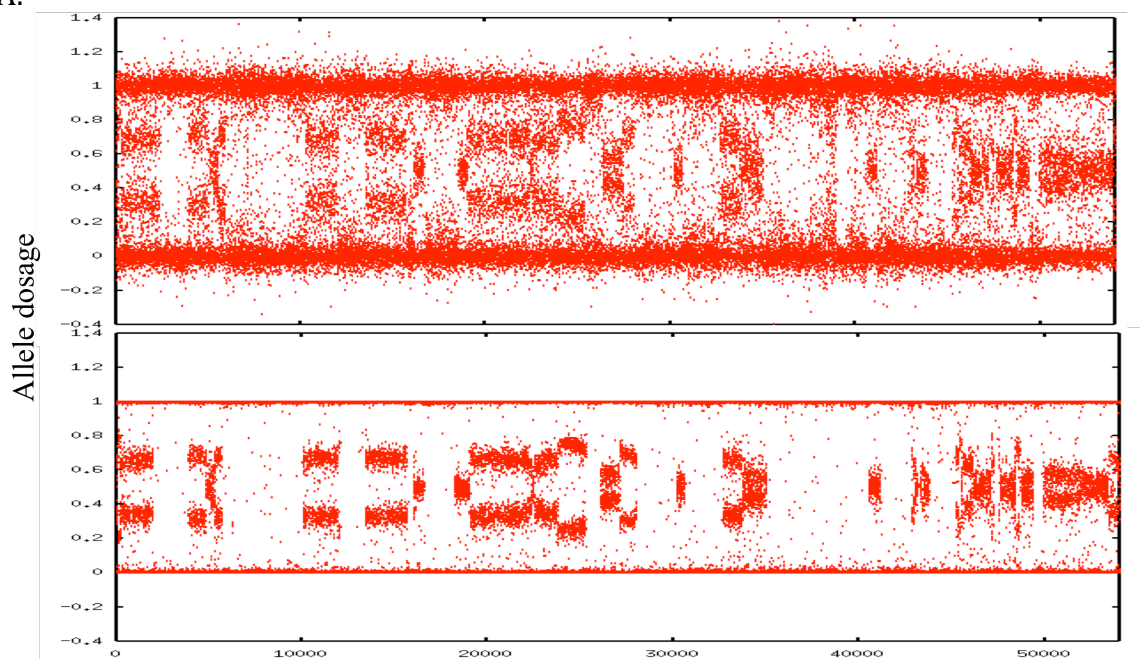

B:

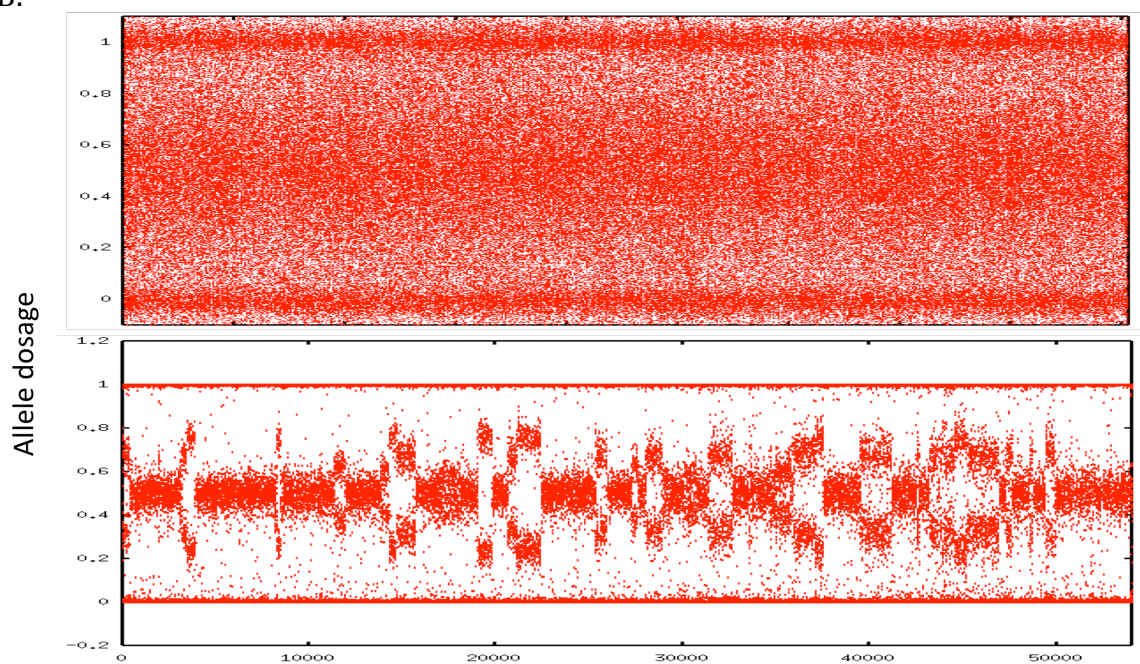

Supplement: Supplementary file 2 — Additional file 2: Figure S1.: Showing allele dosage data generated by SNP microarrays (upper panels) or custom hybridization enrichment followed by next-generation sequencing (lower panels). (A) Frozen samples; (B) FFPE samples. (PDF 2 MB) [file 13058_2014_475_MOESM2_ESM.pdf]

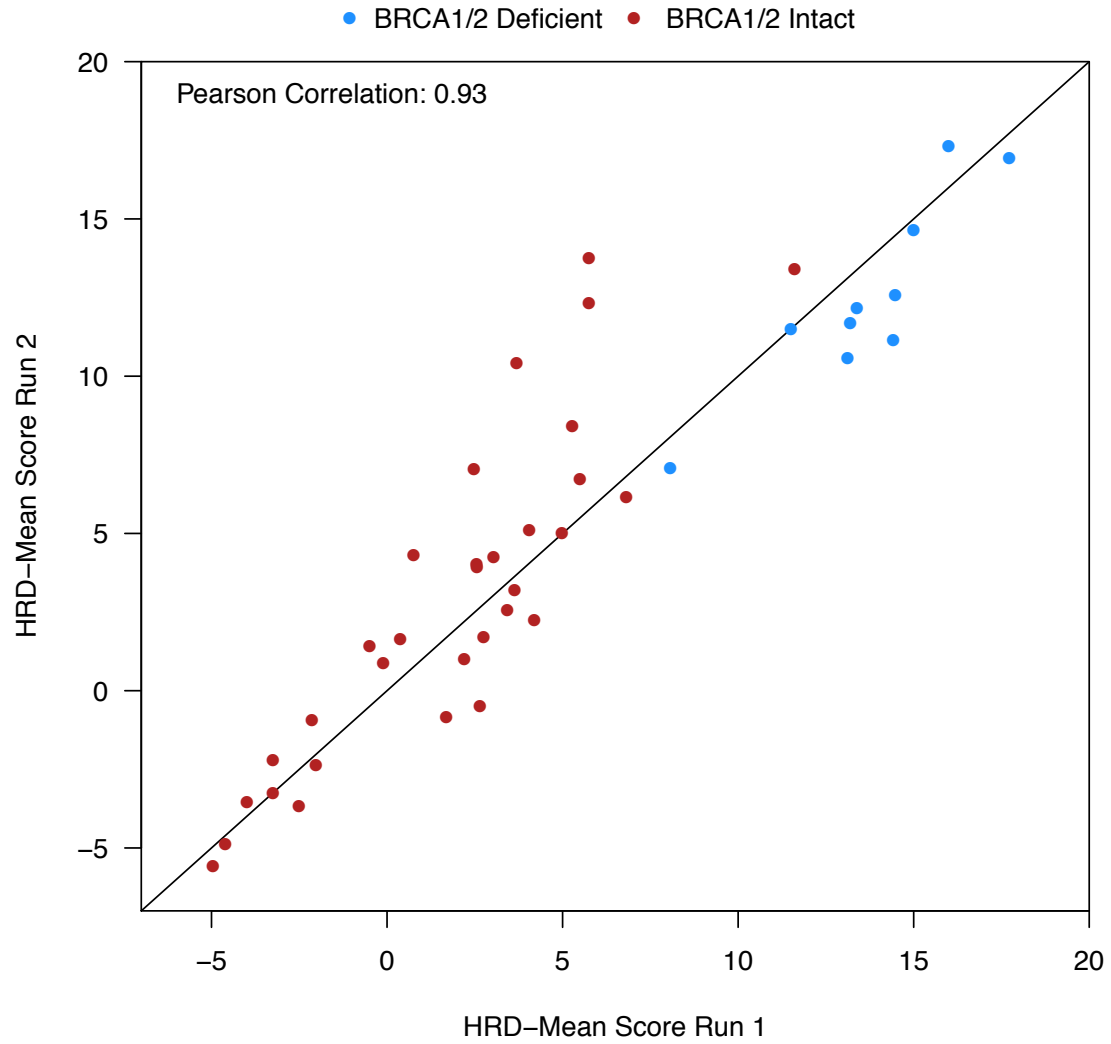

Supplement: Supplementary file 3 — Additional file 3: Figure S2.: Showing correlation between duplicate samples run on the Agilent SureSelect hybridization enrichment assay. Pearson correlation coefficient = 0.93 for HRD-Mean. (PDF 30 KB) [file 13058_2014_475_MOESM3_ESM.pdf]

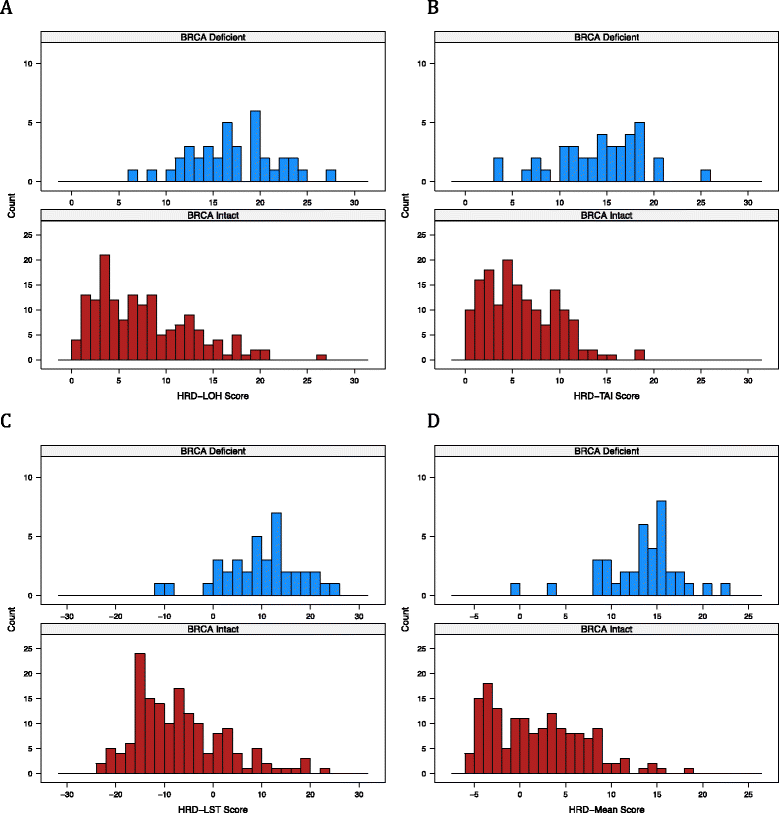

Supplement: Supplementary file 4 — Authors’ original file for figure 1 [file 13058_2014_475_MOESM4_ESM.gif]

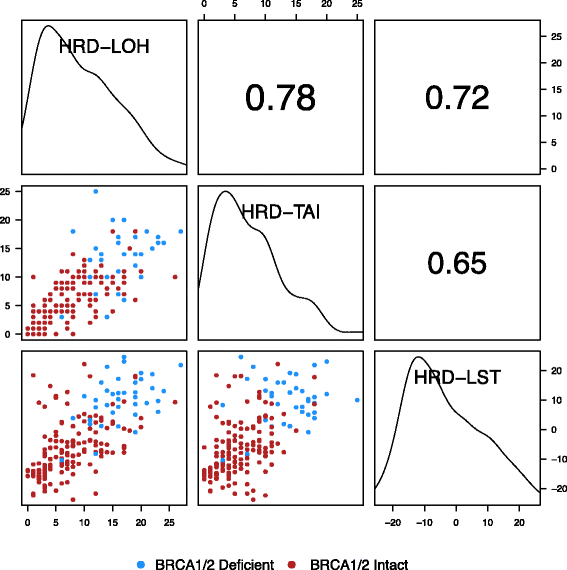

Supplement: Supplementary file 5 — Authors’ original file for figure 2 [file 13058_2014_475_MOESM5_ESM.gif]

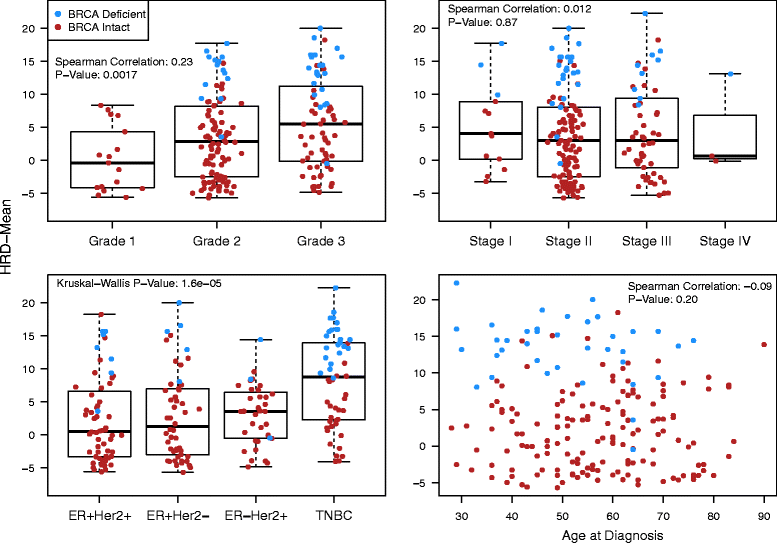

Supplement: Supplementary file 6 — Authors’ original file for figure 3 [file 13058_2014_475_MOESM6_ESM.gif]
